# A Rare Case of ST-Elevation Myocardial Infarction (STEMI) in a Paced Rhythm Meeting Full Sgarbossa Criteria in a 61-Year-Old Male With Complex Percutaneous Coronary Intervention and Post-Infarction Management Challenges

**DOI:** 10.7759/cureus.80192

**Published:** 2025-03-07

**Authors:** Jesse O'Rorke, Greyson Butler, Ramesh Chandra

**Affiliations:** 1 Osteopathic Medicine, Lee Health, Fort Myers, USA; 2 Osteopathic Medicine, Lake Erie College of Osteopathic Medicine, Bradenton, USA; 3 Interventional Cardiology, Lee Health, Fort Myers, USA

**Keywords:** acute coronary syndrome (acs) and stemi, atrial fibrillation (af), congenital unilateral vertebral artery, coronary in stent restenosis, dual lead pacemaker, sgarbossa criteria

## Abstract

In-stent restenosis (ISR) is a common complication following stent implantation and can lead to acute coronary syndrome (ACS), particularly in patients with multiple comorbidities. This condition increases the risk of recurrent angina, myocardial infarction (MI), and the need for repeat revascularization. Although advancements in drug-eluting stents (DES), particularly second- and third-generation models, have significantly decreased the incidence of ISR to about 1-2%, it is still a major concern for high-risk individuals. Managing ACS in the presence of ISR is a complex clinical challenge, requiring careful assessment and intervention. This case report details the presentation, diagnostic challenges, and management of a 61-year-old male with a complex medical history, including atrial fibrillation, chronic obstructive pulmonary disease (COPD), prior MI, and symptomatic bradyarrhythmias treated with a permanent pacemaker. The patient presented with severe chest pain consistent with ST-elevation myocardial infarction (STEMI), later confirmed to result from ISR involving the left anterior descending (LAD) and diagonal arteries. The report highlights the critical role of emergent intervention and advanced diagnostic techniques in managing ISR-related ACS.

A key aspect of this case was the use of Sgarbossa criteria to diagnose STEMI in the presence of an atrial-ventricular paced rhythm. The patient’s EKG demonstrated a perfect score of 10 on the Sgarbossa scale, a rare finding indicative of a high-risk ischemic event. Emergent percutaneous coronary intervention (PCI) was performed, guided by intravascular ultrasound (IVUS), revealing near-complete ISR of the LAD and total occlusion of the first and second diagonal arteries. Successful revascularization was achieved with balloon angioplasty, stent placement, and post-dilation. Post-procedural management required a delicate balance between antithrombotic therapy and bleeding risk, leading to the initiation of dual antiplatelet therapy (DAPT) (It is not specified how long the Cardiology team was going to continue the DAPT in the records).

This report underscores the importance of maintaining vigilance when encountering patients with a history of ISR and prior coronary interventions, emphasizing the need for close monitoring and aggressive management of recurrent ischemic symptoms. Furthermore, it demonstrates the diagnostic value of Sgarbossa criteria in patients with ventricular-paced rhythms and the role of multimodal imaging in guiding optimal PCI strategies. By detailing this patient’s course, this report contributes to the understanding and management of ISR in complex cardiovascular cases, offering insights into optimizing outcomes for high-risk populations.

## Introduction

Acute coronary syndrome (ACS) in the setting of in-stent restenosis (ISR) presents a significant clinical challenge, particularly in patients with extensive cardiovascular and systemic comorbidities. ISR typically presents between three and 12 months post-stent implantation and is often associated with recurrent angina, myocardial infarction (MI), and an increased need for repeat revascularization [1]. ISR that develops within the first year after stent implantation is classified as early ISR, while cases occurring beyond one year are referred to as late ISR. The incidence of ISR has decreased with the advent of second- and third-generation drug-eluting stents (DES), yet it still occurs at a rate of approximately 1-2% after the first year [1,2]. Even higher rates may be observed in patients with diabetes, chronic kidney disease (CKD), and multivessel coronary disease due to increased systemic inflammation and impaired endothelial function [3].

Diabetes and CKD, in particular, contribute to ISR through enhanced neointimal hyperplasia - an excessive proliferation of vascular smooth muscle cells - and neoatherosclerosis, characterized by the formation of de novo lipid-laden plaques within the stent, which accelerates luminal narrowing and increases the risk of late stent failure [4]. While ISR itself is not a direct cause of sudden cardiac death (SCD), it can lead to myocardial ischemia, which in turn can present with angina and may precipitate lethal arrhythmias such as ventricular tachycardia or ventricular fibrillation, ultimately increasing the risk of SCD [5].

This case report details the presentation, diagnostic challenges, and management related to a 61-year-old male with a complex medical history, including atrial fibrillation, chronic obstructive pulmonary disease (COPD), prior MI, and symptomatic bradyarrhythmias treated with a permanent pacemaker. The patient presented with severe chest pain consistent with an ST-elevation myocardial infarction (STEMI), later confirmed to result from ISR involving the left anterior descending (LAD) and diagonal arteries. This report highlights the critical role of timely intervention and advanced diagnostic techniques in managing ISR-related ACS.

This case is significant due to the interplay of multiple comorbidities, the application of Sgarbossa criteria in diagnosing STEMI in the presence of an atrial-ventricular paced rhythm, and the complexity of managing ISR in a high-risk patient. Diagnosing STEMI in the presence of a ventricular-paced rhythm is inherently difficult, as the abnormal depolarization pattern can obscure traditional ischemic changes on EKG. The application of Sgarbossa criteria was essential for identifying ischemia despite the paced rhythm, enabling timely recognition and intervention. The patient's history of ISR and prior coronary interventions underscores the need for close monitoring and aggressive management of recurrent ischemic symptoms. Emergent percutaneous coronary intervention (PCI) guided by intravascular imaging was key in addressing the acute ischemic event, while post-procedural care required balancing antithrombotic therapy with bleeding risk. By detailing the patient's course, this report aims to contribute to the understanding and management of ISR in complex cardiovascular cases, providing insights into optimizing outcomes for high-risk populations.

## Case presentation

A 61-year-old male with an extensive past medical history of anemia, fibromyalgia, atrial fibrillation, prior MI, COPD, congenital absence of unilateral (side not specified in medical records) vertebral artery, Cerebrovascular infarction, bilateral carotid stenosis, symptomatic bradyarrhythmias treated with implantation of a permanent pacemaker, and obstructive sleep apnea presented to the emergency department with a chief complaint of chest pain that radiated to the left arm and jaw with associated shortness of breath that had started one hour before he arrived. The patient reported that he had taken two nitroglycerin tablets at home and his pain had failed to resolve, prompting him to call emergency medical services. He reported that he had experienced nausea and two episodes of vomiting. At admission, the pain was constant and 9/10 in severity. He had a blood pressure of 132/91 mmHg, was hypothermic at a temperature of 35.9 °C, and breathing at 26 respirations per minute. On physical examination by the emergency room physician, the patient was in clear respiratory distress, diaphoretic, with decreased breath sounds at the bilateral lower lung fields with normal S1 and S2 heart sounds without any extra heart sounds or murmurs. He had an initial high-sensitivity cardiac troponin I of 39 ng/L, and a chest radiograph showed no evidence of acute cardiopulmonary abnormality. At this point, the cardiology service was consulted.

**Figure 1:**
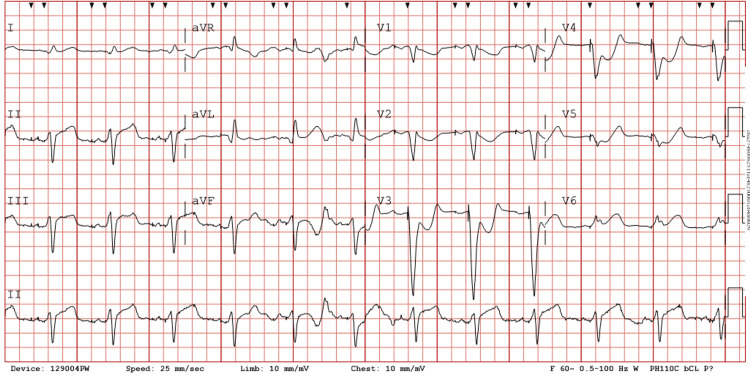
Patient’s EKG on admission The EKG showed concordant ST-segment elevation in lead V6, concordant ST depression in leads V1-3, and excessively discordant ST-segment elevation in leads II, III, and AVF. This finding in the inferior leads is likely due to a wrap around left anterior descending artery. This EKG gave the patient a perfect score of 10 on Sgarbossa’s criteria, a very rare finding. By the criteria, a score greater than or equal to 3 has a specificity of 90% for diagnosing acute myocardial infarction EKG: electrocardiogram

A deeper dive into the patient’s medical history revealed that he had undergone PCI of his LAD artery and diagonal artery in September 2020 for ISR. He went on to reveal that he had been having angina-like symptoms for the last several weeks. He had a nuclear stress test done a few weeks ago, revealing ischemia along the LAD and diagonal artery territory; cardiac catheterization had been ordered and scheduled at that time. He had gone on to develop worsening chest pain the evening of his admission, which had prompted his call to emergency medical services and come to the hospital before his scheduled cardiac catheterization. The cardiology team prepped the catheterization laboratory for emergent transfer and the patient received aspirin and a heparin bolus. He also received nitroglycerin and opioids without much relief from his pain. The EKG showed an atrial-ventricular paced rhythm and STEMI based on Sgarbossa criteria, as shown in Figure 1.

On left heart catheterization, the patient had a mild stenosis of 10-20% in his left main artery, ostial LAD stenosis of 20-30%, long segment stenosis from the proximal to the mid LAD artery of 95-99% severity which was secondary to in-stent stenosis with thrombolysis in myocardial infarction (TIMI) grade 2 flow, indicating partial perfusion. The first diagonal artery was 100% occluded secondary to ISR, which represented complete thrombotic occlusion. Collaterals were noted from the distal LAD artery to the first diagonal artery. The second diagonal was also 100% occluded, secondary to ISR with collaterals from the distal LAD artery. This also represented complete thrombotic occlusion. The left circumflex and obtuse marginal arteries had mild luminal irregularities with TIMI grade 3 flow, indicating complete perfusion. The right coronary artery had mild luminal irregularities as well. There were faint collaterals to septal perforators from the right posterior descending artery. The left ventriculogram showed an ejection fraction of 30% with a large area of apical akinesis with a left ventricular end-diastolic pressure of 19 mmHg. There was no gradient observed across the aortic valve on pullback. 

Percutaneous transluminal coronary angioplasty was performed with a 2.5-mm caliber balloon and then a 60-mm compliant balloon, with significant edge dissection noted. Stent deployment followed in the mid LAD artery. The same stent balloon was used to perform post-dilation along with a 3 x 15-mm noncompliant balloon to perform post-dilation of the stent placed. After this, a stent was deployed in the proximal LAD artery. Following that, a non-compliant balloon was used to perform post-dilation of both of the previously placed stents. An intravascular ultrasound (IVUS) was performed to optimize PCI results. Based on the findings of that ultrasound, another high-pressure inflation of a non-compliant balloon was performed for post-dilation of the proximal LAD artery segment. Following this, the angiographic result was excellent with no perforations, dissections, or thrombus noted post-intervention. The interventional cardiology team recommended uninterrupted dual antiplatelet therapy (DAPT) (interval of time not indicated in the patient's records) following catheterization. 

**Figure 2:**
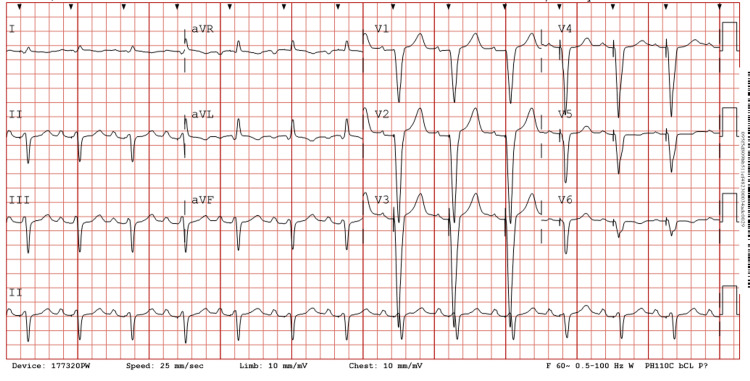
Patient’s EKG following percutaneous coronary intervention, no longer displaying signs of Sgarbossa’s criteria EKG: electrocardiogram

The patient was unable to tolerate antiplatelet therapy by mouth, and hence a cangrelor drip was started until further cardiologist evaluation. The next morning, on evaluation by the cardiology team, the patient had residual 2/10 chest pain that seemed to be pleuritic in nature, most likely due to post-infarction pericarditis (PIP). Colchicine was started at that time. The patient had a high sensitivity cardiac troponin I of 444 and then 25,000. An echocardiogram was done, which showed an ejection fraction of 40-45% with grade 2 diastolic dysfunction with severe hypokinesis of a small area at the apex and distal anterolateral wall. There was moderate hypokinesis of the rest of the anterolateral wall. An EKG was taken that showed an atrial-sensed ventricular paced rhythm with the ventricular pacing tacking p-waves, as shown in Figure 2.

**Figure 3:**
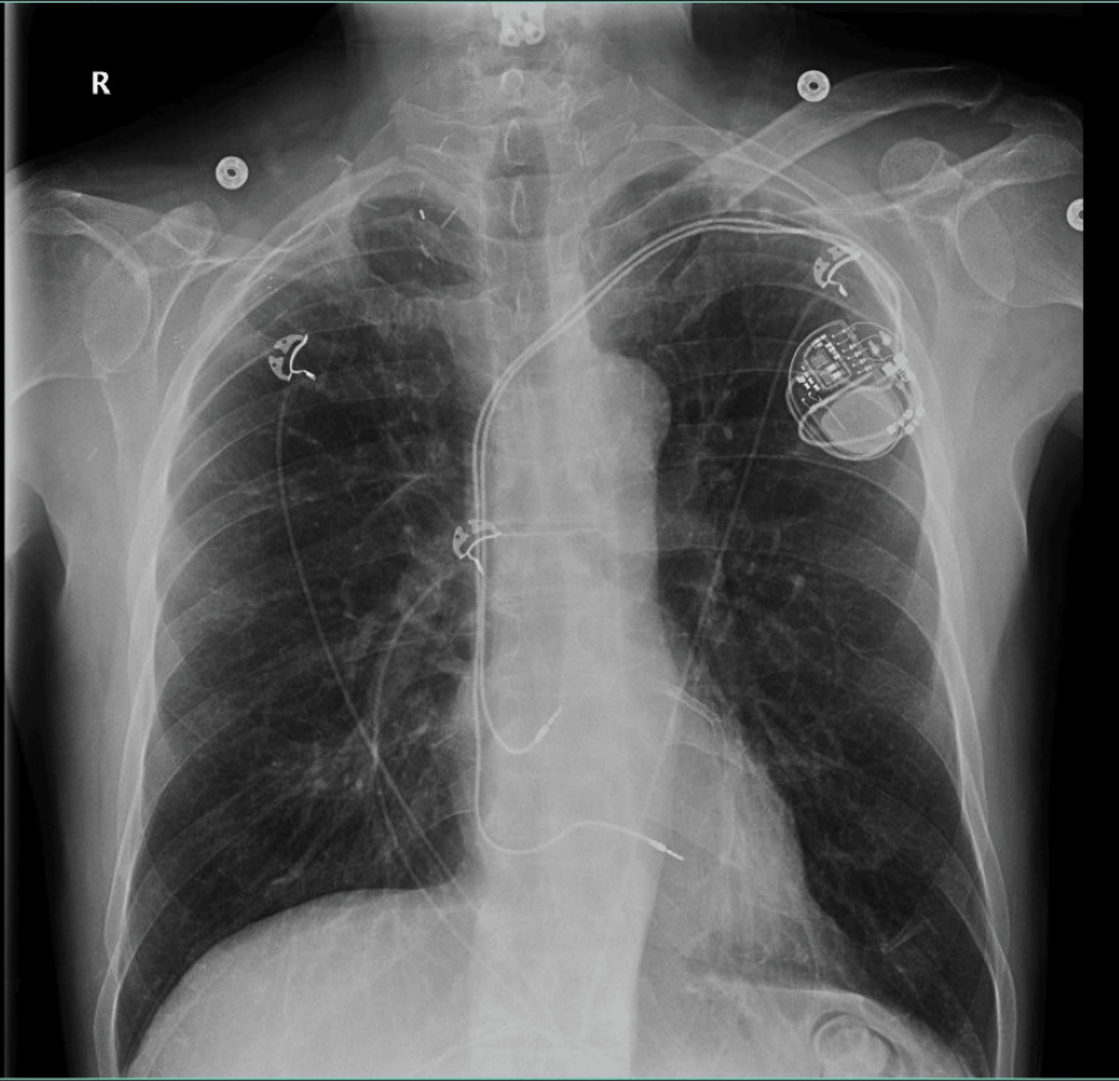
Patient’s chest radiograph The radiograph showed a normal heart size and coronary stent present, a left-sided dual lead pacemaker, a right subclavian stent, and the right clavicular head removed, with normal bone structure and no pneumothorax

At this time, it was recommended that the patient undergo triple therapy with aspirin 81 mg once daily, Eliquis 5 mg twice daily, and clopidogrel 75 mg once daily. It was also recommended that guideline medical therapy for cardiomyopathy be optimized. The patient had no acute overnight events, and, on evaluation by the cardiology team the next morning, his chest pain had completely resolved. Colchicine was discontinued at that time. The patient refused losartan but low-dose spironolactone was added. A chest radiograph was taken, which showed a normal heart size and coronary stent present, a left-sided dual lead pacemaker, right subclavian stent, and the right clavicular head removed, with normal bone structure and no pneumothorax, as shown in Figure 3.

**Figure 4:**
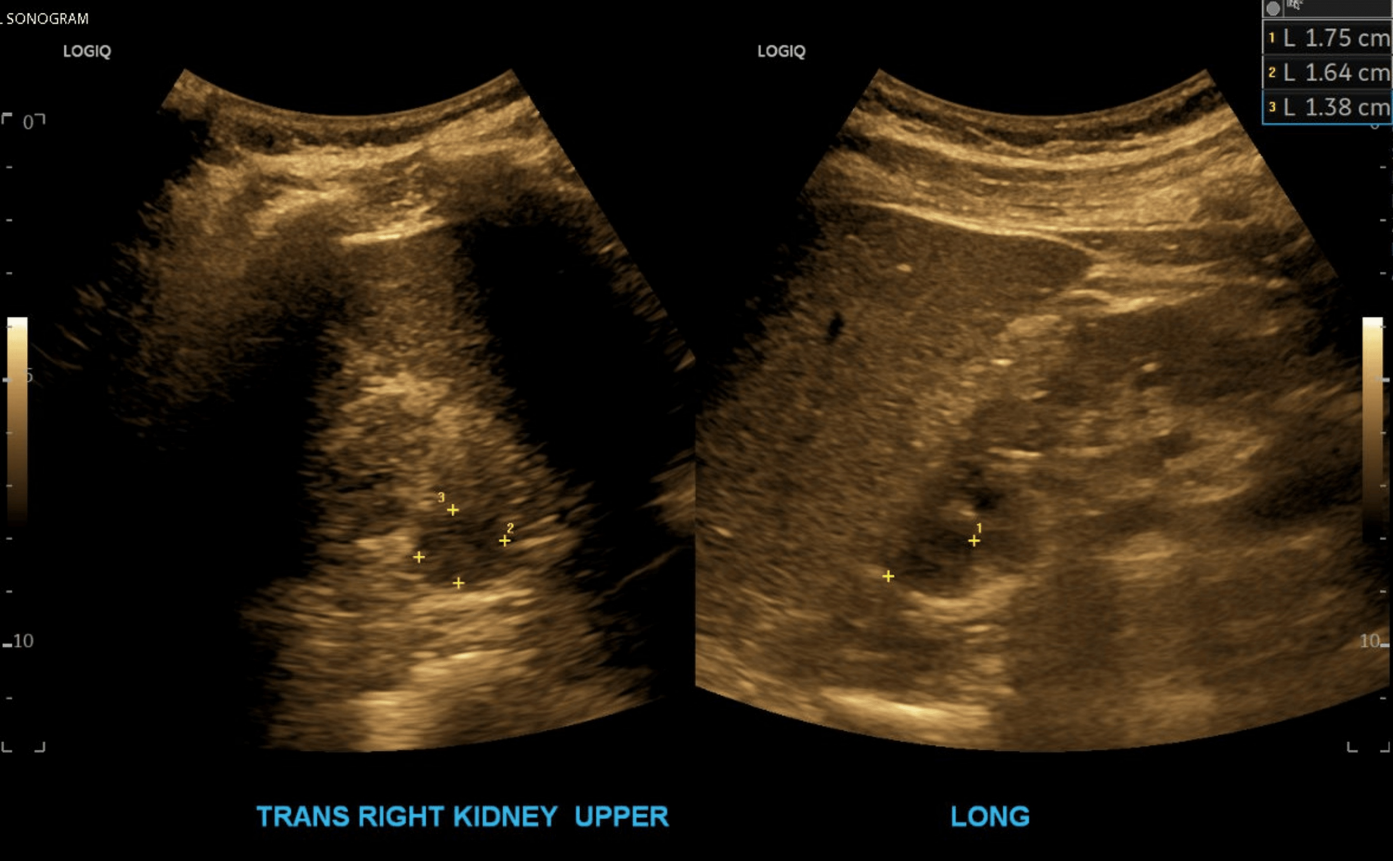
Patient’s renal ultrasound The ultrasound showed a hypoechoic area in the right upper kidney measuring 1.8 x 1.6 cm

A renal sonogram was performed due to the patient’s concurrent acute kidney injury, which showed a hypoechoic area in the right upper kidney measuring 1.8 x 1.6 CM; a non-emergent CT scan with IV contrast was recommended to exclude a renal mass. An image from the ultrasound exam is displayed in Figure 4. The following day, the patient was deemed stable from a cardiac standpoint and he was discharged from the hospital.

Four days later, the patient presented to the emergency room with a chief complaint of an unusual feeling in his chest and left wrist. He was not very specific about his pain and stated that his main concern was the unusual feeling in the wrist. He denied diaphoresis and shortness of breath at that time. He reported mild lightheadedness without syncope on presentation. On physical examination, the patient had unlabored breathing and normal heart sounds. A chest radiograph demonstrated no acute abnormalities; an EKG was performed, which showed findings unchanged from four days prior. High-sensitivity troponin I remained elevated at 2,984 ng/L, likely still reflecting damage from the MI that had occurred four days prior. An echocardiogram was performed, which showed an ejection fraction of 50-55% with borderline left ventricular systolic function and mild apical hypokinesis, and diastolic dysfunction could not be excluded at that time. He was deemed to be in congestive heart failure and started on diuretic therapy. The following day, he was cleared from a cardiac standpoint for discharge with outpatient follow-up with the cardiology team recommended. He was scheduled for a CT scan two weeks after discharge for investigation of his kidney ultrasound findings.

## Discussion

The Sgarbossa criteria are an essential diagnostic tool for identifying acute myocardial infarction (AMI) in patients with left bundle branch block (LBBB) or ventricular-paced rhythms, where traditional EKG patterns of ischemia may be obscured. The criteria assign points based on specific EKG findings: concordant ST elevation >1 mm in leads with a positive QRS complex (5 points), concordant ST depression >1 mm in leads V1-V3 (3 points), and excessively discordant ST elevation >5 mm in leads with a negative QRS complex (2 points) [3]. A total score of ≥3 strongly suggests STEMI, but it is exceedingly rare for a patient to achieve a perfect score of 10 due to the infrequency of all three findings occurring simultaneously. Despite the criteria being highly specific, their sensitivity is relatively low, further emphasizing the rarity of a perfect score [6,7]. Our patient’s EKG not only met the diagnostic threshold for STEMI but demonstrated all three criteria with a perfect score of 10, underscoring the severity of the ischemic event. This rare finding highlights the utility of Sgarbossa criteria in diagnosing STEMI in complex scenarios and reinforces the need for emergent intervention in critical cases.

To address the high specificity but limited sensitivity of the original Sgarbossa criteria, Smith-modified Sgarbossa criteria were adopted. It improved upon the original by replacing the third criterion with an ST/S ratio ≤-0.25, which has shown improved sensitivity from 52% to 91% while maintaining similar specificity [8]. Additionally, unlike the original criteria, which require a cumulative score based on all three criteria, the modified criteria require only one of the conditions to be met for STEMI to be strongly suspected. Because of this improvement, the modified criteria are more effective in cases where ischemic changes may be subtle or where the amplitude of the QRS complex varies significantly [9].

The Smith-modified Sgarbossa criteria were externally validated by Meyers et al., demonstrating superior sensitivity compared to the original Sgarbossa criteria, with only a minor decrease in specificity (93% vs. 90%) [10]. Given this improvement, the modified criteria enhance the detection of STEMI in patients with LBBB or ventricular-paced rhythms without significantly increasing false positives. As a result, the modified criteria are increasingly favored in both high-acuity and non-emergent settings, as they improve diagnostic accuracy without compromising specificity. In this patient the original Sgarbossa criteria effectively identified STEMI; however, the modified criteria may have further reinforced the diagnosis by improving sensitivity to ischemic changes that might otherwise be overlooked.

ISR is a multifactorial process primarily driven by neointimal hyperplasia, characterized by the proliferation and migration of vascular smooth muscle cells and extracellular matrix deposition within the stented segment [11,12]. This pathologic response is initiated by endothelial injury during stent deployment, triggering inflammation and immune cell recruitment that releases growth factors and cytokines to promote smooth muscle cell activation [11-13]. Despite advancements in DES technology, including second-generation DES that releases antiproliferative agents, ISR remains a concern, particularly in high-risk patients. Mechanical factors such as stent underexpansion, malapposition, or fracture can exacerbate ISR by creating areas of turbulent blood flow and promoting neointimal growth [14,15]. Biological factors, such as chronic inflammation, endothelial dysfunction, and late neoatherosclerosis also contribute to the process [13,16-18]. Our patient’s multiple comorbidities, including atrial fibrillation, prior MI, and COPD, likely increased the systemic inflammatory burden and impaired endothelial function, creating a pro-restenotic environment. Furthermore, even with modern DES, ISR occurs in 1-2% of patients annually, underscoring the multifactorial nature of the condition [2].

Interpreting ischemic symptoms in patients with multiple comorbidities and atypical presentations, such as those with ISR, is inherently challenging. Symptoms such as chest pain, dyspnea, and fatigue often overlap with other conditions, such as COPD and atrial fibrillation, as seen in this patient [19,20]. These overlapping clinical features can obscure the diagnosis of ACS. Our patient’s history of recurrent angina and findings from a recent nuclear stress test, which demonstrated ischemia in the LAD and diagonal arteries, were critical in raising suspicion for ISR. Diagnostic tools like coronary angiography and IVUS, which were used in this case, are essential in confirming ISR, understanding its underlying mechanisms, and guiding management [14,15,18,21]. Coronary angiography revealed significant ISR with thrombotic occlusion, while IVUS was instrumental in optimizing stent placement and ensuring lesion coverage during PCI. This case underscores the importance of integrating advanced diagnostic tools into the evaluation of high-risk patients with complex clinical presentations to ensure accurate diagnosis and appropriate treatment.

After stent placement, the management of this patient focused on minimizing the risks of stent thrombosis and recurrent ISR, while addressing the complexities of his medical history. DAPT with aspirin and a P2Y12 inhibitor is the standard of care following stent placement to reduce thrombotic risk [21-23]. However, the patient’s history of recurrent ISR, atrial fibrillation, and other high-risk cardiovascular conditions necessitated a more aggressive approach with triple therapy. He received aspirin, clopidogrel, and Eliquis (apixaban) to address the heightened thrombotic risk. This tailored approach highlights the importance of individualized treatment plans that integrate guideline-directed therapies with patient-specific risk factors to optimize outcomes in complex cases.

PIP is an inflammatory condition of the pericardium that occurs after MI in response to myocardial necrosis. It is typically characterized by pleuritic chest pain, pericardial friction rub, pericardial effusion, elevated inflammatory biomarkers, and sometimes fever [24,25]. In this patient, pleuritic chest pain following PCI raised suspicion for PIP, a diagnosis supported by the absence of ongoing ischemia and the nature of the residual pain. The management involved the use of colchicine, a first-line treatment for pericarditis, which effectively reduced the patient’s symptoms [24]. This case highlights the importance of differentiating PIP from recurrent ischemia and tailoring management to the patient’s unique clinical context to prevent complications such as recurrent pericarditis or pericardial effusion.

The kidney ultrasound findings in this case revealed a hypoechoic area in the right upper kidney measuring 1.8 x 1.6 cm, prompting a CT scan for further evaluation. Common considerations for such a finding include renal cysts, which are typically benign and asymptomatic but may require further evaluation if complex features are present [26]. Another possibility is a renal abscess, particularly in patients with a history of infection or systemic inflammatory conditions [27]. These, however, typically present as flank pain accompanied by fever. Neoplastic processes, such as renal cell carcinoma, must also be considered, especially given the lesion’s focal nature and the patient’s complex medical history. Ischemic injury to the kidney, secondary to hemodynamic instability or embolic phenomena, is another differential, particularly in patients with atrial fibrillation and a history of cardiovascular disease [28]. Further imaging with contrast-enhanced CT or MRI is crucial to distinguish between these possibilities and to guide appropriate management. This case underscores the need for a thorough diagnostic workup to identify the cause of renal abnormalities in patients with multifactorial risks.

Congenital agenesis of one vertebral artery is a rare anatomic variation that holds clinical significance, particularly in the context of cerebrovascular and cardiovascular diseases. The vertebral arteries, which arise from the subclavian arteries, are critical contributors to the posterior circulation of the brain, forming the basilar artery upon their confluence [29]. While typically asymptomatic, the absence of one vertebral artery can become clinically relevant in cases of additional cerebrovascular disease, such as carotid stenosis, as seen in this patient. Ultimately, agenesis may predispose individuals to ischemic events due to reduced overall blood flow and collateral circulation [30-32]. In this patient, the congenital agenesis of a vertebral artery likely compounded the effects of bilateral carotid stenosis and prior cerebrovascular events, limiting the compensatory capacity of the posterior circulation. Recognizing such anatomic variations is crucial for risk stratification and tailored management, particularly in patients with complex vascular pathologies.

## Conclusions

This case highlights the complex interplay between ISR and ACS, particularly in a patient with significant cardiovascular and systemic comorbidities. Despite advancements in DES, ISR remains a clinical challenge that can lead to recurrent ischemic events, as seen in this patient. The application of Sgarbossa criteria in diagnosing STEMI in the presence of an atrial-ventricular paced rhythm in the setting of a permanently implanted pacemaker proved to be a crucial step in identifying the severity of the patient’s ischemic event. Furthermore, emergent PCI with intravascular imaging optimization was essential in achieving successful revascularization in this case. The case also underscores the importance of balancing antithrombotic therapy with bleeding risks in a high-risk patient requiring triple therapy post-intervention.

This report reinforces the need for heightened clinical vigilance in patients with a history of ISR, emphasizing the significance of timely diagnostic evaluation and intervention. Clinicians should maintain a high index of suspicion for ISR in patients presenting with recurrent angina or ACS, particularly in those with PCIs. This case also underscores the importance of post-procedural monitoring and individualized medical therapy to optimize patient outcomes. We hope our findings will contribute to the growing body of knowledge on ISR-related ACS, providing insights into the diagnostic and therapeutic challenges associated with managing complex cardiovascular patients. Future research should focus on refining strategies for ISR prevention, improving long-term stent durability, and optimizing medical management to reduce the recurrence of ischemic events.
